# The medication for pneumocystis pneumonia with glucose-6-phosphate dehydrogenase deficiency patients

**DOI:** 10.3389/fphar.2022.957376

**Published:** 2022-09-07

**Authors:** Ziyu Zhang, Qinhui Li, Xiaoyan Shen, Lankai Liao, Xia Wang, Min Song, Xi Zheng, Yulian Zhu, Yong Yang

**Affiliations:** ^1^ Department of Pharmacy, The First People’s Hospital of Ziyang, Ziyang, China; ^2^ Department of Pharmacy, Sichuan Academy of Medical Sciences & Sichuan Provincial People’s Hospital, School of Medicine, University of Electronic Science and Technology of China, Chengdu, China; ^3^ Department of Medical, Sichuan Academy of Medical Sciences & Sichuan Provincial People’s Hospital, School of Medicine, University of Electronic Science and Technology of China, Chengdu, China; ^4^ Department of Pharmacy, Chengdu Qingbaijiang District People’s Hospital, Chengdu, China; ^5^ Intensive Care Unit, The Third Hospital of Mianyang, Mianyang, China; ^6^ Department of Pharmacy, Ziyang People’s Hospital, Ziyang, China; ^7^ Personalized Drug Therapy Key Laboratory of Sichuan Province, School of Medicine, University of Electronic Science and Technology of China, Chengdu, China

**Keywords:** PCP, G6PD, immunocompromise, oxidant, hemolysis

## Abstract

Pneumocystis pneumonia (PCP) is an opportunity acquired infection, which is usually easy to occur in patients with AIDS, organ transplantation, and immunosuppressive drugs. The prevention and treatment must be necessary for PCP patients with immunocompromise. And the oxidants are currently a typical regimen, including sulfanilamide, dapsone, primaquine, etc. Glucose-6-phosphate dehydrogenase (G6PD) deficiency is an X-linked gene-disease that affects about 400 million people worldwide. The lack of G6PD in this population results in a decrease in intracellular glutathione synthesis and a weakening of the detoxification ability of the oxidants. As a result, oxidants can directly damage haemoglobin in red blood cells, inducing methemoglobin and hemolysis. When patients with G6PD deficiency have low immunity, they are prone to PCP infection, so choosing drugs that do not induce hemolysis is essential. There are no clear guidelines to recommend the drug choice of this kind of population at home and abroad. This paper aims to demonstrate the drug choice for PCP patients with G6PD deficiency through theoretical research combined with clinical cases.

## 1 Introduction

G6PD deficiency is an X-chromosome-linked genotypic disease and was first found by investigating hemolytic development in patients receiving primaquine (Beutler, 1959; Mbanefo et al., 2017). The incidence of G6PD deficiency in southern Chinese cities such as Guangdong, Guangxi, Sichuan, and Hainan province was as high as 4–11% (Jiang et al., 2006; Liu et al., 2012). Except for red blood cells, G6PD may exist in other tissues, but these issues do not seem to be damaged by the lack of the enzyme. The primary purpose of this enzyme in red blood cells is to protect haemoglobin from oxidation. The critical factor in safeguarding haemoglobin from oxidation in red blood cells is glutathione (Prankerd, 1964). As the substrate of glutathione proenzyme, Glutathione protects cells from the toxicity of hydrogen peroxide produced by oxidizing drugs (Cohen and Hochstein, 1963). Due to the lack of G6PD, the cells cannot provide enough nicotine adenine dinucleotides phosphate (NADP) to maintain Glutathione. Glutathione will overused after taking some antioxidants or eating fava beans. The remaining oxygen-free radicals will directly damage red blood cells, leading to cell rupture and hemolytic anaemia. Such oxidant drugs usually include sulfonamides, sulfones, nitrofurantoin, p-aminosalicylic acid, chloramphenicol, and isoniazid (Allen and Wilkerson, 1972). After the onset of the disease, medicine is unuseful for these people but only with relief of symptoms, so avoiding the excessive consumption of glutathione caused by low G6PD activity is the primary measure. The World Health Organization (WHO) recommended that people in areas where the prevalence of G6PD deficiency is more than 5% should be routinely tested for enzymes at birth. However, the coverage rate of this detection is low. There are still many G6PD deficiency positive patients with hemolytic anaemia after using oxidant drugs. There is sufficient evidence that patients with G6PD deficiency are prohibited from using the following seven drugs: dapsone, methylthioninium chloride (methylene blue), nitrofurantoin, phenazopyridine, primaquine, rasburicase, and tolonium chloride (Youngster et al., 2010).

Pneumocystis pneumonia is an opportunistic infection, its prevention and treatment are essential. The dangerous factors for PCP infection are severe immune deficiency for instance HIV, long-term use of glucocorticoids, tumour, transplantation and severe malnutrition. In that case, the mortality rate exceeds 90% (Hughes et al., 1975; Dei-Cas, 2000; Herrag et al., 2010; Gilroy and Bennett, 2011; Weyant et al., 2021). Sulfonamides and sulfoxides commonly used for PCP patients, and may cause serious adverse reactions in patients with G6PD deficiency. Therefore, it is difficult to choose drugs when patients with such enzyme deficiency are complicated with PCP infection. This article will focus on the prevention and treatment of PCP drugs and analyze the impact of these drugs on patients with G6PD deficiency. To reduce the harm of drugs to such patients, providing the best choice for the prevention and treatment of PCP in patients with G6PD deficiency.

## 2 G6PD deficiency

G6PD is not specific to red blood cells. It is a housekeeping enzyme that exists in almost all human cells. The monomer of G6PD is composed of 515 amino acids with a molecular weight of about 59 kDa (Cappellini and Fiorelli, 2008). G6PD can catalyze the oxidation of glucose-6 - phosphate (G6P), convert NADP into NADPH, and provide the reduction capacity for body cells in the form of NADPH (Cappellini and Fiorelli, 2008). NADPH can promote the production of reduced glutathione (GSH), thereby reducing the oxidative stress response of oxidants to body cells (Tsai et al., 1998). In most human cells, NADPH is a crucial component in many biosynthesis processes, including the synthesis of fatty acids, cholesterol, and steroid hormones, and the generation of deoxyribose. G6PD plays an important role in reducing hydrogen peroxide and oxygen free radicals and maintaining hemoglobin and other erythrocyte proteins. In most cells, in addition to G6PD, there are many enzymes catalyzed dehydrogenase reactions to produce NADPH, so even G6PD deficiency cells do not lead to short of NADPH. However, NADPH production in red blood cells is entirely different. The difference is that, with red blood cell differentiation and other enzyme inactivation, NADPH has no other source than the pentose phosphate pathway (Luzzatto et al., 2016). Therefore, red blood cells defend against oxidative stress depending on G6PD and are more vulnerable to G6PD deficiency than other cells. The defects of G6PD are primarily incomplete, and the NADPH produced by the remaining G6PD activity is sufficient to maintain RBC operation but usually short-lived. At this time, if there is exogenous oxidant stimulation, G6PD-deficient red blood cells will not produce enough NADPH and GSH for consumption, which will cause damage to haemoglobin or other proteins, and eventually, lead to red blood cell rupture and hemolysis.

### 2.1 Genetic characteristics of G6PD deficiency

The G6PD gene is composed of 13 exons of 515 amino acid protein subunits. Except for the binding site between NADP substrate and G6P substrate, each subunit has a closely bound NADP molecule (Au et al., 2000). G6PD deficiency is caused by G6PD gene mutation, resulting in varying degrees of enzyme deficiency and protein variation, which is related to various clinical subtypes. The most usual clinical diseases are neonatal hyperbilirubinemia and acute hemolytic anemia, and exogenous drug use is the main cause.

In the case of G6PD deficiency, the genetic defect is located in the subtelomere region of the long arm of the X chromosome and is also affected by X chromosome inactivation. (Beutler et al., 1962). X linkage has an essential effect on the genetic characteristics of G6PD deficiency. There are only two genotypes in males: hemizygote normal and half zygote G6PD deficiency, while females, have three genotypes: homozygote normal, homozygous deficient, and heterozygote (Cappellini and Fiorelli, 2008). It is generally considered incorrect that the incidence of G6PD deficiency is higher in males than in females since homozygote female are less likely than hemizygote men. Still, there are many more heterozygous females, from the basic principles of population genetics (Hardy-Weinberg equilibrium). These heterozygotic females usually show that half of the red blood cells are G6PD deficient, and half of the red blood cells are G6PD normal. Some heterozygous female patients offer a normal state, and some show a disease state similar to homozygous, which has apparent clinical significance (Rinaldi et al., 1976).

### 2.2 Epidemiological characteristics of G6PD deficiency

The geographical distribution of G6PD deficiency is extensive, with the frequency peak of infections found in Africa, the Middle East the Mediterranean region, and Asia; however, due to recent migration, this disease occurs in the United States and Northern Europe. The prevalence of the disease is highest in Africa, Asia, the Middle East, Latin America and the Mediterranean. Among them: Kurdish Jews 60–70%, Sardinia 4–35%, Nigeria22%, Thailand17%, Greece6%, South China6%, India3%. The lack of G6PD affects black Americans, of which up to 24% are carriers, and about 10% are affected by black males (Harcke et al., 2019). It is found that the distribution of G6PD deficiency is similar to that of malaria endemic areas. This indirect evidence suggests that G6PD deficiency is resistant to malaria (Ruwende and Hill, 1998), but it does not prove that malaria selects for the gene that causes G6PD deficiency. Natural selection seems responsible for the higher mortality rates of children from malaria in endemic areas. By comparing the incidence of malaria, parasiteia levels, or the severity of malaria in children with G6PD normal and deficiency, studies conducted in Africa and other regions showed that the lack of G6PD appeared to have a protective effect on severe malaria (Rockett et al., 2014; Luzzatto et al., 2016), but the results need more data to support.

According to the law of natural selection, the genetic characteristics associated with the X-linked G6PD gene tend to be stable if both males and females with protective genes increase adaptability (Luzzatto, 2012). However, there is no evidence that population genes have evolved into G6PD deficiency in malaria-prone areas. Although so much current research shows that G6PD deficiency protects against malaria, especially falciparum malaria, the evidence is still insufficient. Therefore, the idea that G6PD deficiency is a protective factor for malaria requires more research data.

### 2.3 Clinical manifestations of G6PD deficiency

Patients with G6PD deficiency usually have no obvious clinical manifestations but can lead to disease only under exogenous stimulation. In infants with G6PD deficiency, neonatal jaundice (NNJ) risk is higher. There is not enough evidence for this phenomenon, but this may be the most common cause of NNJ in countries where G6PD deficiency is widespread. Serious complications may occur in semi-zygotic boys and girls with G6PD deficiency (Doxiadis et al., 1964). Another clinical manifestation of acute hemolysis is often caused by exogenous substances, including fava beans or some drugs, such as chloroquine, sulfanilamide, naphthalic acid, etc.

Red blood cells (RBC) may rupture after eating fava beans in G6PD deficiency patients. Ruptured RBC can lead to a sharp drop in haemoglobin, acute hemolysis, and hemoglobinuria. A particular glycoside in fava beans causes this phenomenon and can occur at any age, but it is more usual and dangerous in childhood (Luzzatto et al., 2016). Studies have shown that patients with G6PD are more likely to develop sepsis, so treatment for patients with G6PD deficiency needs to be more cautious (Spolarics et al., 2001). Infectious diseases are also one of the risk factors for hemolysis. So, it is challenging to distinguish whether the cause of hemolysis is disease or drugs. When a patient with G6PD deficiency is co-infected, the choice of medicine will be critical (Youngster et al., 2010).

## 3 Pneumocystis pneumonia

### 3.1 Definition

PCP can lead to aggravation of diseases and even death in patients with immunocompromised, especially with HIV infection (Huang et al., 2006; Herrag et al., 2010; Huang, 2011). Initially, Plasmodium Cysticercosis was initially classified as protozoans because of their morphological characteristics similar to those of protozoans and their sensitivity to antiprotozoal drugs. Because its cytoderm composition and nucleotides are identical to fungi, they have recently been classified as fungi. The earliest molecular biological evidence also suggests that Plasmodium cysticercosis is a fungus (Santamauro et al., 2002; Lu and Lee, 2008).

### 3.2 Clinical manifestations

The symptoms and signs of pneumocystis pneumonia are atypical and are often mistaken for infections caused by other bacteria or viruses (Weyant et al., 2021). The common symptoms of PCP include dyspnea, fever, and hacking cough, and there are other atypical symptoms, such as chest pain, hemoptysis, hypoxia, and diffuse dry rale during an examination (Singhal et al., 2005; Fujii et al., 2007; Thomas and Limper, 2007). The clinical manifestations of HIV complicated with PCP infection are different from those of other causes of immune dysfunction. The course of the PCP in patients with HIV is often longer, usually manifested as a hidden course with symptoms; some studies showed the duration could be as long as 28 days (Catherinot et al., 2010). Patients with immunodeficiency without HIV tend to have more severe symptoms and a higher risk of respiratory failure and death (Krajicek et al., 2008; Weyant et al., 2021).

### 3.3 Characteristics of the disease

It may not be abnormal or accompanied by strange respiratory sounds in the initial chest examination. Still, later, the disseminated rale and pulmonary shadow will be severe if not treated. The typical manifestations are diffuse interstitial syndrome on x-ray chest film and diffuse bilateral ground-glass shadow on CT, mainly in the perihilar inferior areas. Other features include focal patchy parenchymal consolidation, cysts, solid nodules, pneumothorax, and some cavity and honeycomb lesions (Santamauro et al., 2002; Fujii et al., 2007; Thomas and Limper, 2007). Zaman et al. proposed that elevated serum lactate dehydrogenase (LDH) levels are closely associated with PCP infection. In AIDS patients, the absolute level greater than 450 IU may suggest the infection of pneumocystis pneumonia, while normal levels indicate a lower likelihood of PCP infection (Zaman and White, 1988). However, LDH is not a specific indicator, especially in some potential malignant tumors or patients with liver dysfunction. Whether or not the people affected by HIV or the level of LDH is within the normal range, these patients may develop pneumocystis pneumonia (Santamauro et al., 2002). Sputum induction is a susceptible method for diagnosing pneumocystis pneumonia in laboratory tests, with a sensitivity between 55 and 95% for PCP in HIV-infected persons (Santamauro et al., 2002). Bronchoalveolar lavage fluid is another practical test for PCP in immunosuppressed patients, and the test has almost 100% sensitivity and peculiarity. In addition, the current methods used to detect PCP-positive microorganisms are immunofluorescence (IFL), cytology, polymerase chain reaction (PCR), or silver staining, in which PCR is more sensitive but cannot distinguish colonization from infection (Santamauro et al., 2002; Azoulay et al., 2009; Catherinot et al., 2010; Wilson et al., 2011).

### 3.4 Susceptible population

Generally, Pneumocystis mainly causes infection in immunocompromised patients but can colonize in individuals with standard immune systems and spread to patients with immune impairment (Ponce et al., 2010). Among them, the causes of immune deficiency are HIV, glucocorticoids, and cellular immune deficiency. *Cancer* (especially haematological malignancies), hematopoietic stem cell transplantation (HSCT), or solid organ transplantation acceptor are the leading causes. Following, connective tissue diseases, systemic diseases, rheumatism, severe immunodeficiency, and severe malnutrition also play a role (Dei-Cas, 2000; Herrag et al., 2010; Sadanand, 2011; Weyant et al., 2021). In patients without HIV infection, the most critical risk factor for PCP was glucocorticoids and cell-mediated immunity deficiencies (Sepkowitz et al., 1992; Sepkowitz et al., 1995).

### 3.5 Treatment programmes

According to the symptoms, signs, and chest radiography of PCP patients, the disease status is divided into three grades: mild, moderate, and severe. Despite other medications for pneumocystis pneumonia, trimethoprim-sulfamethoxazole (TMP-SMX) remains the recommended first-line treatment for mild to middle infections, which has good oral bioavailability (Singhal et al., 2005; Carmona and Limper, 2011). Intravenous or oral administration can achieve appropriate serum levels in patients without impaired gastrointestinal function. TMP-SMX (15 mg/kg every 6–8 h) was routinely administered according to renal function. As for non-critical patients who can take oral medication, two double dosage tablets every 8 h are recommended (Goto and Oka, 2011). Sulfoxide combined with trimethoprim, primaquine combined with clindamycin, and atovaquone is the second-line treatment options for mild to moderate PCP patients. The first-line drug for patients with severe infection is still TMP-SMX. And primaquine combined with clindamycin, caspofungin combined with TMP-SMX, and intravenous injection of Pentamidine is the second-line drugs. Besides, In some cases, methotrexate plus calcium folinate can be used as a rescue treatment for PCP (Huang et al., 2006; Calderón et al., 2010; Rouyer et al., 2015). Glucocorticoids can reduce pulmonary inflammation caused by pulmonary cysticercosis for patients with severe PCP. It significantly prevents oxidative deterioration, mortality, and intubation in the first 7 days of HIV treatment (50 percent reduction) (Briel et al., 2005). For non-HIV patients with severe PCP, daily doses of prednisone greater than or equal to 60 mg were more effective than lower doses (Pareja et al., 1998).

### 3.6 Preventive measures

In patients with HIV, the count of CD4 T cells is a helpful marker and classifies the risk of PCP. TPCP patients need Primary prevention when the count of CD4 cells is lower than 200/mm^3^. However, in patients without AIDS, there were no valuable markers for monitoring immune status (Santamauro et al., 2002). TMP-SMX is the first selection to prevent PCP; sulfoxide is the second-line drug to prevent PCP, which is banned in G6PD enzyme deficiency patients (Bellamy, 2008). Atovaquone is a suspension that only fatty foods can promote its absorption. It has been widely studied in the human immunodeficiency virus population and small-scale trials of solid organ transplantation recipients. It can be used as a second-track drug to prevent PCP. Compared with TMP-SMX, sulfoxide, or Atovaquone, inhaled injection of Pentamidine is less effective and should be regarded as a third-line drug to prevent PCP. In the study of HIV patients, clindamycin combined with pyrimidine was neither less effective than TMP-SMX nor sulfoxide or pentamidine (Davey and Masur, 1990; Goto and Oka, 2011; Rouyer et al., 2015; Brakemeier et al., 2018).

## 4 The effect of drugs in pneumocystis pneumonia with G6PD deficiency

The most severe consequence of patients with G6PD deficiency is that the red blood cells of some patients will cause oxidative damage and acute hemolysis under drugs, acute diseases, and certain foods (such as broad beans). If patients can avoid using some medications to reduce oxidative stress exposure, the incidence of hemolysis may be significantly reduced. Some oxidant medicines for the prevention and treatment of PCP can lead to acute hemolysis in patients with G6PD deficiency. In the following, we will analyze the drugs used for PCP prevention and treatment one by one to evaluate the hemolysis risk of these drugs in patients with G6PD deficiency.

### 4.1 The influence of oxidants

At present, sulfonamides are the main recommended drugs in the first line for PCP, among which TMP-SMX is the optimal choice for these patients. The main alternatives include sulfoxide, trimethoprim, primaquine, clindamycin, etc. However, sulfoxide-trimethoprim and clindamycin-primaquine regimens are prohibited in Use in G6PD deficiency (Warren et al., 1997; Castro, 1998; Sadanand, 2011). These common oxidant drugs may cause a risk for acute hemolysis in G6PD deficiency. Hemolytic anaemia caused by G6PD deficiency is a self-constraint process; sometimes, anemia may not be apparent. The WHO classifies G6PD deficiency into five grades based on the wide range of enzyme activity in genotypes and heterozygotes: grade 1 showed severe deficiency, accompanied by chronic non-spherical hemolytic anaemia, grade 2 severe deficiency (enzyme activity was 1–10%), grade 3 moderate deficiency (the range of enzyme activity is 10–60%), grade 4 regular (enzyme activity 60–150%) The activity of grade 5 was enhanced (>150%) (WHO Working Group, 1989). Although the enzyme activity is different, oxidant drugs can cause hemolytic anaemia in various stages of enzyme deficiency. Several drugs are associated with acute hemolysis in G6PD deficient population, such as Primaquine, Sulfanilamide, Sulfapyridine, TMP-SMX, dapsone, Nitrofurantoin, Co-trimoxazole (Beutler, 1964; Beutler, 1996).

#### 4.1.1 Sulfonamides

TMP-SMX, as the most optimized selection for PCP, can reduce mortality and intubation rates. Compared with controls, A Cochrane meta-analysis reports a 91% reduction in the incidence of PCP and an 83% reduction in mortality (Hughes et al., 1975; Maschmeyer et al., 2016). At the same time, metabolic disorders, drugs, and hepatitis are also factors of hemolysis in G6PD deficiency. Patients with PCP infection who use sulfonamides may increase the danger of hemolysis due to the influence of ailment and drugs. TMP-SMX has been associated with severe side effects of medicine source hemolytic anemia due to lack of G6PD activity (Frank, 2005). In an early study, 75% of patients with G6PD deficiency developed hemolysis after treatment with sulfonamides. However, sulfonamides are not contraindications for all patients with G6PD. In black women, there was a low risk of severe hemolysis after therapy with sulfonamides in G6PD deficiency (Norden et al., 1968). In the Chan et al. Study, ten infants with G6PD deficiency were treated with TMP-SMX at a dose of 5–10 mg/kg, and the daily dose is about 30–50 mg. Before and 5 days after treatment, we reviewed the haemoglobin, hematocrit, reticulocyte count, and blood smear and found no signs of hemolysis in the infant. We found an experiment result; even if the G6PD deficiency showed the same activity as the G6PD enzyme, it could lead to differences in drug metabolism due to liver and kidney function changes or some uncertain metabolic characteristics, or other coexisting diseases (Chan et al., 1976). Whether or not these sulfonamides cause severe hemolytic anaemia, the World Health Organization and many studies have banned their use in the G6PD deficiency population. Above all, it is best to prevent G6PD deficiency patients from choosing such drugs and selecting other medicines.

#### 4.1.2 Primaquine

Primaquine, combined with clindamycin as an alternative treatment for PCP, is an antiparasitic agent used primarily to prevent and treat malaria. A controlled study of primaquine for safety and tolerance showed that the average hematocrit of G6PD deficiency patients taking primaquine decreased significantly on the 7th, 8th, and 9th day (*p* = 0.015, 0.027, 0.048) (Krudsood et al., 2006). Some early tests demonstrated that primaquine at a daily dose caused intravascular hemolysis in glutathione-deficient red blood cells, with severity associated with glutathione, and suggested that ascorbic acid might alleviate such hemolysis (Greenberg and Wong, 1961). So, we must be careful about using primaquine in patients with a G6PD deficiency.

Several studies have shown that primaquine has dose-dependent hemolysis: a higher dosage of primaquine may lead to significant clinical hemolysis in G6PD heterozygotic women. A prospective study of 801 malaria patients showed that in Thailand, where the epidemic variation of G6PD deficiency was relatively mild, many sufferers with G6PD deficiency could not tolerate large doses of primaquine. In a subject with G6PD deficiency, high doses of primaquine may be contraindicated, but standard doses of primaquine with careful monitoring of hematocrit may also be a treatment option (Silachamroon et al., 2003). It has been found that giving primaquine twice a week provides more hemolytic effect than once a week. In contrast, the control group without G6PD deficiency does not cause anaemia (Zipursky et al., 1965; Tine et al., 2017).

Low doses of primaquine are safer than high doses of primaquine: the WHO recommended combined therapy of a single dose of 0.25 mg/kg. Standard artemisinin is safe in treating acute uncomplicated Plasmodium falciparum malaria, whether or not patients have G6PD deficiency. Other tests also showed a lower risk of hemolysis after daily administration of primaquine with a single dosage of 0.25 mg/kg (Eziefula et al., 2014; Bancone et al., 2016; Mwaiswelo et al., 2016; Bastiaens et al., 2018; Dysoley et al., 2019). In a systematic evaluation of primaquine in the therapy of malaria, the higher dose of the remedy, the higher the rish of anaemia in G6PD deficiency compared with placebo. However, there was no significant difference in haemoglobin decrease on the 7th day compared with placebo at low doses such as 0.25 mg/kg (Uthman et al., 2017).

The hemolysis caused by primaquine and chloroquine in Caucasians or Asians with g6pd deficiency has not been thoroughly studied. Still, enzyme deficiency is more severe in these groups than in blacks, and even given low doses of drugs can cause more severe hemolysis (Brewer and Zarafonetis, 1967). The severity of hemolysis in G6PD deficiency flocks relies on enzyme deficiency, the accumulated dose, drug exposure time, and other agents such as infection, age, and haemoglobin (Hb) concentration (Cappellini and Fiorelli, 2008).

#### 4.1.3 Dapsone

Dapsone is a sulfone antibiotic that can prevent pneumocystis pneumonia in patients with sulfonamide allergy. The primary adverse reaction was hemolytic anaemia caused by sulfone drugs. The hdroxylamine is a toxic metabolite, the leading cause of hemolysis. It is mainly produced by the metabolism of cytochrome P450 enzymes after the absorption of dapsone in the gastrointestinal tract into the blood system through the portal vein to the liver. It generates free radicals, which cause haemoglobin damage and hemolytic anaemia forasmuch as glutathione consumption. In patients with G6PD deficiency, these hydroxylamine metabolites consume large amounts of glutathione, rapidly deplete its stores and increase the chance of hemolytic anaemia (Grossman and Jollow, 1988; Jollow et al., 1995; Zhu and Stiller, 2001). In addition, the antioxidants vitamin C or other vitamins cannot neutralize the metabolites produced by dapsone, and such antioxidants do not reduce the incidence of hemolytic anaemia.

There are some adverse reactions associated with dapsone in transplant patients. In individuals with G6PD deficiency, children who received sulfoxide treatment for malaria developed hemolytic anaemia more frequently than those who received the treatment of artemisinin (29%) (Van Malderen et al., 2012). Animal experiments showed that individuals lacking G6PD showed a tripling sensitivity to hemolytic anaemia induced by sulfoxide (Grossman et al., 1995). The Enzyme activity detection is a routine test for G6PD deficiency, but molecular analysis of female heterozygotes may be required. We found a Greek woman whose G6PD enzyme activity was detected to decrease and developed severe acute hemolysis following initiation of dapsone therapy (Lee and Geetha, 2015). A multicenter randomized controlled study of thousands of people showed signs of hemolysis in almost all G6PD deficient children receiving sulfoxide, with normal erythrocyte morphology before treatment and a significant decrease in haemoglobin after treatment (Pamba et al., 2012). A study in North America reported that in a population with stem cell transplantation (SCT), dapsone had a higher risk of hemolysis than dapsone and TMP-SMX in preventing PCP. Although most of the patients who received sulfoxide prophylaxis were negative for G6PD activity fluorescence screening tests, there is still existed hemolysis induced by dapsone (Zhu and Stiller, 2001). Another trial also showed that dapsone had a higher risk of hemolysis than TMP-SMX (87 vs. 0%, *p* = 0.001) (Olteanu et al., 2012). Four patients switched from TMP-SMX to dapsone in renal transplants due to allergy to sulfonamides and subsequently developed methemoglobinemia. So, early identification and discontinuation of sulfoxide were essential (Salim et al., 2017). Another study showed that 46% of 26 renal transplants using sulfoxide also developed methemoglobin (Mitsides et al., 2014). A randomized controlled clinical trial of complicated falciparum malaria in Africa also suggested that the risk of haemoglobin decline in patients with G6PD deficiency was even higher in the Chlorproguanidine - dapsone group than in the sulfadoxine-pyrimethamine group (Alloueche et al., 2004). Several studies have found that the incidence of methemohemoglobin induced by dapsone is higher than that of TMP-SMX. This may be due to the differentiation in the formation, disposal, virulence and detoxification of TMP-SMX and dapsone hydroxylamine metabolites (Reilly et al., 1999). The above studies showed that sulfoxide is more likely to consume reduced glutathione in red blood cells as an oxidant, so we do not advise patients with G6PD deficiency to choose sulfoxide to prevent or treat PCP.

### 4.2 The effect of non-oxidants

As for the prevention and treatment of PCP, gene screening and enzyme activity examination are the first step in patients with G6PD deficiency. Then, we recommended different drugs according to the degree of enzyme deficiency. We should conduct Long-term follow-ups in patients with a serious degree of G6PD deficiency, and tell the one and their families about the food and drugs which is prohibited and cautious to use. Regardless of the degree of enzyme deficiency in patients, we should also give the appropriate medication guidance to patients to prevent hemolytic diseases. In this paper, we summarize several PCP prophylaxes and therapeutic drugs for the patients with G6PD deficiency.

#### 4.2.1 Atovaquone

Atovaquone is traditionally known as a broad-spectrum antiparasitic drug. It is similar to the coenzyme Q, which inhibits the electron transport chain by preventing ubiquinone from binding to cytochrome B (Baggish and Hill, 2002). HIV-infected patients with TMP-SMX or sulfoxide tolerance, especially patients with G6PD deficiency (also known as favism), can be treated with Atovaquone. Although Atovaquone is not as effect availability as TMP-SMX in the PCP, its main advantage is oral administration, tolerable side effects, and fewer adverse reactions, so it is the replacement therapy for mild to moderate PCP (Weyant et al., 2021). Among solid organ transplant recipients with regular G6PD activity, dapsone had more significant haemoglobin reduction and drug discontinuation rates compared with Atovaquone (Hedvat et al., 2021). Douzinas et al. reported A clinical case in a G6PD deficiency patient with severe PCP infection have completely cured with Atovaquone (Douzinas et al., 2010). However, Atovaquone cannot be used in combination with rifampin, azanavir and weilun, because these drugs can reduce its blood concentration.

#### 4.2.2 Pentamidine

Pentamidine is an aromatic diamidine with a wider scope of antimicrobial effects. And it has an optimal investigation in antigenic animals such as trypanosomiasis and leishmaniasis. It is given that it has a broad antibacterial mechanism. It can reduce polyamine synthesis by inhibiting the ornithine carboxylase and binding the Trypanosoma motor DNA and RNA to reduce polyamine synthesis. It can also inhibit polymerase impairment of ribosome function and the synthesis of nucleic acid and protein. The mechanism of action remains unclear. There is currently sufficient evidence for the use of pentamidine in the treatment of PCP, and we recommend intravenous injection as a major alternative to TMP-SMX for moderate to severe PCP (Calderón et al., 2010). The effective rate of atomizing Pentamidine in preventing the first attack of PCP in HIV- infected patients was 60–70%. There were no apparent side effects except cough (Hirschel et al., 1991). A systematic review of the second-line treatment of PCP suggests that Pentamidine may be more commonly used in patients with severe PCP but may lead to more deaths than TMP-SMX due to the adverse risk of spraying amidine (Benfield et al., 2008). In addition, a retrospective research of adverse reactions caused by pentamidine treatment of PCP showed that nephrotoxicity, abnormal blood glucose, hepatotoxicity, hyperkalemia, and hyperamylasemia accounted for 80%, and hemolytic anaemia was not reported (O'Brien et al., 1997). So, Pentamidine is a relatively safe option in patients with G6PD deficiency.

#### 4.2.3 Clindamycin

Clindamycin is one of the Lincomycin derivatives and has good antimicrobial activity against Gram’s positive bacterium and Gram-negative anaerobes. The primary mechanism is to inhibit the ribosome of 50s and prevent the synthesis of proteins. In protozoa, the target of clindamycin is a parasite-specific organelle (a plastid organelle), which reduces toxin production in bacterial staphylococci and streptococci. However, the mechanism of its use in the treatment of PCP is not precise (Fichera and Roos, 1997). Clindamycin combined with primaquine as a second-line treatment is more effective than Pentamidine in patients with TMP-SMX intolerance, according to a study (Kim et al., 2009). In a meta-analysis by Smego et al., the success rate of clindamycin combined with primaquine in the treatment of PCP was 92% higher than that of Atovaquone and Pentamidine (Smego et al., 2001). There are no reports of hemolytic anaemia caused by clindamycin in patients with G6PD deficiency. However, clindamycin and primaquine are often combined to prevent and treat PCP. As an oxidant, primaquine has a particular effect on G6PD deficiency patients. However, studies in recent years have reported that low-dose primaquine has less risk of hemolytic anaemia in G6PD deficiency (Eziefula et al., 2014; Bancone et al., 2016; Mwaiswelo et al., 2016; Uthman et al., 2017; Bastiaens et al., 2018; Dysoley et al., 2019). Clindamycin combined with low dose primaquine may be a potential solution for PCP patients with G6PD deficiency, but more clinical trials are needed to validate this regimen.

#### 4.2.4 Echinocandins

Echinocandins (such as caspofungin, micafungin, etc.) are a new class of antifungal drugs targeting β (1, 3)-D-glucan. Because there is β (1, 3)-D-glucan in the cell wall of cysticercosis, Echinocandins can kill pneumocystis by inhibiting the synthesis of β (1, 3)-D-glucan. Some animal experiments showed that the drug was influential in the animal model of PCP (Furuta et al., 1997; Kamboj et al., 2006). There is no wide prospective analysis on the management of PCP with Echinocandins, and many case reports or cohort studies reported the clinical data as a remedial measure for the failure of TMP-SMX (Annaloro et al., 2006; Hof and Schnülle, 2008; Armstrong-James et al., 2011). Herbert Hof reported a 60-year-old patient with PCP complicated with Wegener granulomatosis. After failing to respond to TMP-SMX treatment, the patient switched to intravenous caspofungin at a load dosage of 70 mg and a maintenance dose of 50 mg/day. Symptoms improved 3 weeks later (Hof and Schnülle, 2008). The success of this case suggests that echinocandin may be a reliable candidate for the treatment of PCP, which deserves more research and attention.

Similarly, ten patients who could not tolerate first-line therapy used caspofungin in a European retrospective clinical study of HIV with PCP. Eight patients were successfully treated (one patient died of pneumothorax, and one died of lymphoma). Who suggested that caspofungin was effective as a salvage treatment (Armstrong-James et al., 2011). There is less data on Echinocandins in G6PD deficiency and PCP. Still, some clinical cases presented that an HIV-infected patient diagnosed with PCP and G6PD deficiency achieved successful results after 3 weeks of treatment with Anidulafungin when TMP-SMX was disabled (Chang et al., 2018).

### 4.3 Assisting effect

#### 4.3.1 Glucocorticoids

Glucocorticoids as immunosuppressants may increase the chance of Pneumocystis pneumonia infection. Higher doses of glucocorticoids have been reported in some studies related to higher mortality in patients with PCP (Ando et al., 2019). So, is glucocorticoid appropriate as a treatment for PCP patients with G6PD deficiency? We found the answer through some clinical reports. For patients with severe HIV-associated PCP, adjunctive corticosteroids are an advocate in PCP patients whose arterial oxygen partial pressure is lower than 70 mmHg. Early use of glucocorticoids can reduce pulmonary inflammation and edema, improve respiratory status, reduce the risk of respiratory failure, and improve the severe adverse reactions caused by sulfonamides (Bozzette et al., 1990; Gagnon et al., 1990; Wazir and Ansari, 2004). A meta-analysis showed that adjuvant corticosteroid therapy could reduce the hazard of death in the HIV-associated PCP population with hypoxia. Still, there is no evidence that adjuvant glucocorticoid therapy was effective for mild PCP (Briel et al., 2005). Moreover there are limited data to support the action of adjuvant corticosteroids in the therapy of non-HIV-associated PCP, and further research is needed to explain the role of corticosteroids in PCP (Injean et al., 2017). So, for patients with severe PCP, early glucocorticoid use seems beneficial. However, there is little evidence that auxiliary glucocorticoid is effective for mild PCP.

## 5 Summary

There are several different degrees according to enzyme activity in G6PD deficiency. Patients with a mild enzyme activity deficiency may not have any adverse reactions. Still, patients with a moderate and severe lack of enzymes may have hemolytic anaemia and other severe consequences after using oxidants, such as sulfanilamide, dapsone and primaquine. Therefore, it is challenging to select medicines for PCP when the activity of G6PD is unknown. If medical conditions permit, we recommend measuring the G6PD enzyme activity before using oxidant drugs. The patient’s hematocrit and the clinical manifestation of hemolysis should be monitored regularly during oxidant drugs (Todd et al., 1994; Belfield and Tichy, 2018). For patients with G6PD deficiency, according to the enzyme activity, the most effective measure is to avoid exposure to oxidant drugs. Choose atovaquone and pentamidine, which have more evidence in the alternative plan. Other drugs, such as clindamycin, Echinocandins, and glucocorticoid, can be used as a single or combined remedy.

## 6 Discussion

The screening of G6PD deficiency in China is still not routine surveillance, and its pathogenesis belongs to X chromosome linkage incomplete dominant inheritance. Male patients usually show a significant decrease in enzyme activity, while female patients are mainly heterozygotes. The range of G6PD enzyme activity varies widely, and the diagnosis is difficult, which challenges the choice of drugs. If such patients use oxidant drugs, they may have serious consequences: such as sulfonamides, which can lead to dangerous or fatal hemolytic anaemia (Bernstein and Lorincz, 1981; Norby et al., 1981). For G6PD deficiency, primaquine should be avoided or carefully used under the supervision of experts (Kovacs and Masur, 2009; Lalloo et al., 2016). In addition, even after taking the standard dose of primaquine, the specific volume of blood cells began to decrease at 2–4 days, reached the lowest level at 8–12 days, and developed reticulocytosis 4–6 days later, accompanied by elevated serum bilirubin, as well as a series of symptoms of fatigue, grief, fever, and jaundice (Reinke et al., 1995). Therefore, we generally do not recommend oxidant-type drugs for patients with G6PD deficiency who need to prevent or treat PCP.

Pneumocystis pneumonia is the most common life-threatening infection in immunocompromised populations. A study reported that in the absence of prevention, the incidence of PCP in solid organ transplant recipients was 6.8–22%, so it is recommended to receive PCP prevention at least 6 months after organ transplantation (Al-Raisi et al., 2009). People living with HIV, recipients of hematopoietic stem cell transplantation (HCT) or solid organ transplantation, cancer patients (especially patients with hematological malignancies), and people with low immunity receiving glucocorticoids, chemotherapy drugs and other immunosuppressive drugs are all at high risk for PCP infection. The National Comprehensive *Cancer* Network (NCCN) Clinical Practice Guidelines in Oncology recommend that in acute lymphoblastic leukemia, the prevention of PCP should run through the entire anti-leukemia treatment process. We successfully implemented PCP prevention for a 4 - year-old child with G6PD deficiency and acute T-lymphocyte leukemia. The patient was screened in Sichuan Provincial People’s Hospital at birth and found to have decreased G6PD enzyme activity and diagnosed with G6PD deficiency. The boy was diagnosed with acute T-cell lymphoblastic leukemia in September 2021. He had been treated with vincristine, daunorubicin, thiopurine, and dexamethasone, and intrathecal injection of methotrexate, cytarabine, and dexamethasone to prevent central leukemia. According to the NCCN guidelines, patients with acute lymphoblastic leukemia who were treated with chemotherapy drugs were at high risk of PCP infection. We implemented measures to prevent PCP in the boy. Due to the lack of G6PD, clindamycin 5 mg/kg was selected as the second-line regimen to prevent PCP thrice a day. During the treatment of leukemia for half a year, the infection of PCP was successfully avoided in children, indicating that clindamycin can be used as a preventive drug for such patients, with good safety and no adverse reactions such as hemolytic anemia.

Combined with clinical cases and previous experience, we suggest that for patients with G6PD deficiency, it is recommended that red blood cells, haemoglobin, reticulocytes, etc., should be monitored regularly before or after the use of sulfonamides or sulfoxide and other oxidant drugs, because some adverse reactions such as hemolytic anaemia may subside when sulfonamides stop taking drugs (Bernstein and Lorincz, 1981). Not clear about the inspection indicators will affect our diagnosis of G6PD deficiency and may have adverse effects on the future when using oxidant drugs. For patients with G6PD deficiency, regardless of their enzyme activity, our recommendation is to avoid the application of control for PCP and to choose other drugs as alternative treatments; we recommend six options as follows:

1) Atovaquone is rarely used in China, making it difficult to obtain these drugs, especially in many primary hospitals in China. However, it is still an adequate substitute for the PCP’s prophylactic and therapeutic. For patients with G6PD deficiency, we recommend that Atovaquone 750 mg be taken twice a day for oral treatment or 1500 mg once daily for prevention (Dennis and Kasper, 2019).
2) Pentamidine is a recommended alternative drug for PCP in the current guidelines, including 3∼4 mg/kg, given intravenously once a day, or 300 mg monthly atomization therapy to prevent PCP (Dennis and Kasper, 2019). The efficacy is worse than that of Atovaquone, TMP-SMX, or dapsone, but its occurrence of hemolytic anaemia in patients with G6PD deficiency is minor. So, it is also our recommended drug.
3) In recent years, there have been reports and studies on the adverse reactions to haemoglobin reduction of primaquine. Whether or not patients suffer from G6PD deficiency, many studies have confirmed that there is no a significant difference in the adverse reactions of hemolytic anaemia between patients with G6PD deficiency and ordinary patients when primaquine is used with a low dose of 0.25 mg/kg/d. Therefore, we suggest that without other better options, patients with G6PD deficiency can be given low dose primaquine 0.25 mg/kg/d under the supervision of a doctor. Not more than 7 days of use, and need to monitor haemoglobin, red blood cells, reticulocytes, and so on.
4) In our case, Clindamycin is also a good choice about it successfully prevented PCP infection in a 4-year-old child. Regarding clindamycin treatment, we generally do not recommend a single drug treatment because its single drug use effect is poor. As the PCP alternatives treatment, we recommend clindamycin 300–450 mg Every 6 h, or 600 mg, Every 6–8 h (Dennis and Kasper, 2019)combined with primaquine 0.25 mg/kg/d. Pay close attention to the use of related blood indicators.
5) Caspofungin is an alternative therapy for PCP. However, there are few reports, and several literature reports on the treatment of PCP of sulfonamides are not suitable for successful examples. In addition, Carpofungin is metabolized slowly by hydrolysis and n-acetylation and spontaneous chemical degradation without the inhibition of the CYP system. According to the treatment experience, our recommended dose is 70 mg/d for the first time, followed by 50 mg/d, and the course of treatment depends on the patient’s remission (Annaloro et al., 2006; Hof and Schnülle, 2008; Armstrong-James et al., 2011). It may be a wonderful choice for PCP patients with G6PD deficiency who cannot tolerate sulfonamides, dapsone, primaquine and other drugs.
6) Although the evidence is limited, prednisone or methylprednisolone 40 mg once or twice a day for 5 days in patients with moderate to severe PCP is recommended (Bozzette et al., 1990; Gagnon et al., 1990). A reasonable method is to treat patients by taking at least 20 mg of prednisone a day for more than 1 month (Kovacs and Masur, 2009).

There were differences in the risk of drug use in patients with G6PD deficiency due to genetic polymorphism. Although a few case reports suggest that some patients have a low risk of hemolysis after medication, we still should monitor during treatment. Our suggestions come from clinical studies and case reports. We have not found randomized controlled trials or meta-analyses to demonstrate our views, so our opinions have some limitations. We expect that there will be more clinical studies on the prevention and treatment of PCP infection in G6PD patients in the future to provide the basis and guarantee for the safety of drug use in these patients.
